# The association of early linear growth and haemoglobin concentration with later cognitive, motor, and social–emotional development at preschool age in Ghana

**DOI:** 10.1111/mcn.12834

**Published:** 2019-06-13

**Authors:** Maku E. Ocansey, Seth Adu‐Afarwuah, Sika M. Kumordzie, Harriet Okronipa, Rebecca R. Young, Solace M. Tamakloe, Brietta M. Oaks, Mary Arimond, Kathryn G. Dewey, Elizabeth L. Prado

**Affiliations:** ^1^ Program in International and Community Nutrition, Department of Nutrition University of California Davis California USA; ^2^ Department of Nutrition and Food Science University of Ghana Accra Ghana; ^3^ Department of Nutrition and Food Sciences University of Rhode Island Kingston Rhode Island USA; ^4^ Intake ‐ Center for Dietary Assessment, FHI 360 Washington DC USA

**Keywords:** cognitive development, haemoglobin concentration, linear growth, postnatal, prenatal

## Abstract

It is important to identify the periods during childhood when exposure to environmental risk factors results in long‐term neurodevelopmental deficits. Stunting and anaemia may be sensitive indicators of exposure to such risks. In a prospective cohort enrolled before birth, we investigated the association of developmental scores at 4–6 years with (a) birth length and linear growth during three postnatal periods and (2) haemoglobin (Hb) concentration at three time points. Children were participants in a follow‐up study of a randomized controlled trial of nutritional supplementation in Ghana. At 4–6 years, cognitive, motor, and social–emotional developments were assessed using standard tests adapted for this population. We estimated the associations of length‐for‐age *z*‐score (LAZ) at birth and postnatal linear growth (*n* = 710) and Hb (*n* = 617) with developmental scores in regression models, using multistage least squares analysis to calculate uncorrelated residuals for postnatal growth. Cognitive development at 4–6 years was significantly associated with LAZ at birth (β = 0.12, 95% CI = 0.05, 0.19), ΔLAZ from 6 to 18 months (β = 0.16, 95% CI = 0.04, 0.28), and Hb at 18 months (β = 0.13, 95% CI = 0.06, 0.20), but not with ΔLAZ during 0–6 months, ΔLAZ from 18 months to 4–6 years, Hb at 6 months, or Hb at 4–6 years. No evidence of associations with motor or social–emotional development were found. These results suggest that in similar contexts, the earlier periods prior to birth and up to 18 months are more sensitive to risk factors for long‐term cognitive development associated with LAZ and Hb compared with later childhood. This may inform the optimal timing of interventions targeting improved cognitive development.

**Key messages**

- In low‐ and middle‐income countries, the ages at which linear growth faltering and anaemia occur may be related to the likelihood of poor neurodevelopment.
- In a prospective study using longitudinal data on linear growth during four phases of development and Hb concentrations at three postnatal time points, LAZ at birth and ΔLAZ from 6 to 18 months as well as Hb at 18 months were associated with cognitive development at age 4–6 years.
- In this and similar settings, the identified critical periods for long‐term cognitive development may help inform the optimal timing of interventions targeting improved cognitive development.

## 1 INTRODUCTION

In the first 2 years of life, neurodevelopmental processes such as neurogenesis, synaptogenesis, and myelination occur rapidly (Prado & Dewey, 2014), making infants particularly vulnerable to biological and environmental risk factors that affect cognitive, behavioural, and motor developments. Children who experience linear growth faltering and anaemia tend to have lower developmental scores during this period (Adair et al., 2013; Lozoff et al., 2006). For children living in low‐ and middle‐income countries (LMICs), linear growth faltering is caused by multiple risk factors, such as prenatal undernutrition (Christian et al., 2013), recurrent infections, and illnesses as well as energy‐protein malnutrition and micronutrient deficiencies due to inadequate diets (Prendergast & Humphrey, 2014), which co‐occur within a complex interplay of psychosocial factors such as poverty, low maternal education, inadequate care and nurturing from caregivers, and lack of learning opportunities (Black et al., 2013). Anaemia may be caused by nutritional factors, including inadequate intakes of bioavailable iron and other micronutrients and impaired absorption of nutrients, as well as nonnutritional factors such as intestinal blood loss due to infections (Balarajan, Ramakrishnan, Özaltin, Shankar, & Subramanian, 2011). The timing of faltering in linear growth and low Hb during childhood may be sensitive indicators of the timing of exposure to these environmental risk factors. The main objective of the present study was to identify the periods during development and the developmental domains that are most sensitive to long‐term insults from such risk factors, using the timing of faltering in length‐for‐age z‐scores (LAZ) and low Hb concentration as markers of the timing of exposure.

At least 12 studies in LMICs have assessed the relative association of linear growth measured at multiple time points throughout childhood with developmental or school outcomes after age 2 years using a statistical approach that accounts for correlations between repeated linear growth measures. Nine of these studies found stronger and more consistent associations of early linear growth (before age 1 or 2 years) as compared with linear growth during later childhood with later cognitive and school outcomes (Adair et al., 2013; Cheung & Ashorn, 2010; Crookston et al., 2013; Glewwe, Jacoby, & King, 2001; Hamadani et al., 2014; Kowalski et al., 2018; Kuklina, Ramakrishnan, Stein, Barnhart, & Martorell, 2006; Li, Digirolamo, Barnhart, Stein, & Martorell, 2004; Pongcharoen et al., 2012). The other three studies found the opposite pattern (Fink & Rockers, 2014; Gandhi et al., 2011; Teivaanmäki et al., 2017). These studies have focused on cognitive scores or educational achievement, with only one assessing motor (Kuklina et al., 2006) and none assessing social–emotional development. Although several studies have examined relationships of Hb concentration during early life with long‐term developmental outcomes (Ai, Zhao, Zhou, Ma, & Liu, 2012; Aubuchon‐Endsley et al., 2011; Aubuchon‐Endsley et al., 2013; Eilander et al., 2010; Grantham‐Mcgregor & Ani, 2001; Jianghong, Adrian, Venables, & Mednick, 2004; Olney et al., 2009; Su et al., 2016), only one study to our knowledge reported independent associations with Hb during specific periods of childhood by adjusting for Hb at other time points (Aubuchon‐Endsley et al., 2013).

In the present study we addressed these research gaps using longitudinal data from a randomized controlled trial in Ghana (Adu‐Afarwuah et al., 2015), to identify the critical periods during which long‐term development in three domains—cognitive, motor, and social–emotional behaviours–—is sensitive to insults from risk factors associated with linear growth faltering and low Hb. We included two separate indicators of exposure to environmental risk factors in our analyses: Hb concentration and changes in linear growth during different periods of childhood. We adjusted for several potential confounding factors associated with growth, Hb, and development. Given that the predominant pattern in previous studies (9/12) was stronger associations in earlier than later childhood, our first hypothesis was that linear growth during early life (prenatal, birth to 6 months, and 6–18 months) but not during later childhood (18 months to 4–6 years) would be positively associated with developmental outcomes at 4–6 years. Secondly, we hypothesized that child Hb concentration during early childhood (6 months and 18 months) would be positively associated with developmental outcomes at 4–6 years even when controlling for concurrent Hb concentration. The specific time points examined were constrained by the available data from the main trial and follow‐up study.

## 2 METHODS

### 2.1 Study design and participants

This study was a follow‐up of children who participated in the International Lipid‐based Nutrient Supplement (iLiNS) DYAD randomized controlled trial in Ghana. The iLiNS DYAD‐Ghana trial was conducted from 2009 to 2014 and randomized pregnant women to three intervention arms: (a) lipid‐based nutrient supplements provided to women during pregnancy and for 6‐month postpartum, and to their infants from 6 to 18 months of age; (b) maternal multiple micronutrient supplements provided to women during pregnancy and 6‐month postpartum; and (3) maternal iron and folic acid provided to women during pregnancy and calcium placebo tablet during 6‐month postpartum. Children in the latter two groups received no direct supplement during infancy. Details of the original trial are presented elsewhere (Adu‐Afarwuah et al., 2015).

The follow‐up study was conducted when children were 4–6 years old. All children and their mothers who participated in the original study were invited to participate. The institutional review boards of the University of California, Davis, and the Ethics Committees for the College of Basic and Applied Sciences at University of Ghana and the Ghana Health Service approved the protocol for the follow‐up study. Written informed consent was obtained from parents or caregivers for their and their child's participation before data collection. Of the 1,222 children eligible for follow‐up, 79% (*n* = 966) were enrolled. In the present analyses, we created two subsamples. The first subsample included all children for whom complete data were available on LAZ at all four time points (LAZ at birth, 6 months, 18 months, and 4–6 years), the 14 covariates with <2% missing data, and at least one of the three developmental outcome measures (*n* = 710, the “LAZ subsample”). The second subsample included all children for whom complete data were available on Hb at all three time points (6 months, 18 months, and 4–6 years), the 14 covariates with <2% missing data, and at least one of the three developmental outcome measures (*n* = 617, the “Hb subsample”). For the remaining three covariates with 3–5% missing data, we imputed missing values using multiple imputation, as described below.

### 2.2 Data collection

Maternal and household information including maternal age, parity, education, household assets, and food insecurity were collected at enrolment into the original trial by trained fieldworkers using a questionnaire.

Child length at birth, 6 months, and 18 months and height at 4–6 years were measured to the nearest 0.1 cm using a stadiometer by trained anthropometrists who were standardized for anthropometric data collection both in the original trial and at follow‐up. The details of the methods of measurement are presented elsewhere (Adu‐Afarwuah et al., 2015; Kumordzie, Adu‐Afarwuah, Arimond, Young, & Adom, 2019). The measurements were converted into *z*‐scores according to World Health Organization standard procedures (WHO Multicentre Growth Reference Study and de Onis, 2006).

At 6 and 18 months of age, trained laboratory personnel using standard safety measures collected blood by venipuncture and immediately measured blood Hb using a digital Hemocue (HemoCue model 301, AG, Switzerland). At 4–6 years of age, the capillary blood sampling procedure was used and Hb concentration was measured using a digital Hemocue (201+ model).

#### 2.2.1 Developmental assessments

Neurobehavioral development was assessed at follow‐up in three domains: cognitive, motor, and social–emotional function. Details of the methods are described in Table 1. Fine motor function was assessed by the NIH Toolbox 9‐hole pegboard test (Bauer & Zelazo, 2014). Social–emotional competence was assessed by caregiver interview using the total difficulties score on the Strengths and Difficulties Questionnaire (Goodman, 1997; Goodman & Goodman, 2009). Cognitive function was assessed by several measures from the following subdomains: language, executive function, pre‐academic skills, visuospatial attention, and declarative memory (see the supporting information for details).

**Table 1:** Motor, cognitive, and social–emotional measures of assessment^a^

Developmental domain	Developmental test	Test description and scoring
Motor		
Fine motor	NIH Toolbox 9‐Hole Pegboard	We recorded the time required for children to accurately place and remove nine plastic pegs from a pegboard, first with the dominant hand, followed by the other hand. The score was the average time in seconds taken to complete the task with each hand.
Cognitive		
Language ability	NEPSY‐II body part naming and identification	Children were asked to say aloud or point to body parts on a line drawing of a person or on their own body. The two scores were the sum of body parts correctly named and identified.
	NEPSY‐II comprehension of instructions	Children were instructed to point to a picture for example “show me a puppy that is big and blue and happy.” The score was the number of items indicated correctly.
Visuospatial ability	Block design	Children were asked to copy increasingly complex patterns of models built by the instructor, using wooden block in 30 s. The score was the number of structures correctly copied within the time limit.
Declarative memory	Paired‐associate learning and recall task	Children were first taught new words for pictures of eight objects and were asked to point to them as the instructor mentioned them aloud. They were later asked to recollect the words learned after a delay of median (interquartile range) 7 (6–11) min. We calculated the score as the average number of correct responses on a set of eight learning trials and two delayed recall trials.
Executive function		
Visual selective attention	Visual search test	Based on the NEPSY subtest, children were asked to identify all instances of a target picture (chicken or kitten) printed on a page with other distracter pictures as quickly as possible in 2 min. The score was the total time in seconds per correct target item identified.
	IDELA head/toes test	Children were asked to inhibit the normal response to touch their head when instructed to do so, by touching their toes instead. This was repeated five times interchanging the touch head or toes instruction in a particular order. The score was the sum of correct responses.
Pre‐academic skills	Parent's Evaluation of Developmental Status (PEDS) preacademic subscale	In 14 items, children were asked to perform skills such as counting, reading aloud words, or identifying letters of the alphabet. The score was the total of correct responses.
Social–emotional		
Psychosocial and prosocial characteristics	Strengths and Difficulties Questionnaire (SDQ)	Parents or caregivers were asked during an interview to describe their child's behaviour within the past 6 months, based on a set of 25 questions divided between five scales: (a) emotional symptoms, (b) conduct problems, (c) hyperactivity/inattention, (d) peer relationship problems, and (e) prosocial behaviour. Responses were scored on a Likert scale ranging from 0 to 2 (not true, somewhat true, and certainly true, respectively). Attributes 1 to 4 were summed up to generate a “total difficulties score.”

a References are in the supporting information.

For each developmental test score, we calculated *z*‐scores based on the distribution of scores in our sample in 3‐month age bands. For the motor domain, the average *z*‐score for the dominant and nondominant hands was calculated. For cognitive development, an overall cognitive factor score was calculated as the first factor of a factor analysis using the principal‐axis factoring method including seven outcome measures: body part naming and identification, comprehension of instructions, preacademic skills, visual search, head‐toe, block design, and paired associate scores. The first factor was the only factor with an eigenvalue greater than 1 (eigenvalue = 2.9) and accounted for 79% of the variance in the cognitive scores.

#### 2.2.2 Additional covariates measured at follow‐up

The amount and quality of nurturing care available from the home environment at age 18 months were assessed using the Family Care Indicators Interview (Kariger et al., 2012) and at 4–6 years using the Early Childhood version of the Home Observation for the Measurement of the Environment (HOME) Inventory (Caldwell & Bradley, 2003). To be consistent with previous waves of data collection in this cohort, maternal depressive symptoms were assessed at 4–6 years using the Edinburgh Postnatal Depression Scale. The Edinburgh Postnatal Depression Scale is a valid tool even among women who have not recently given birth (Cox, Chapman, Murray, & Jones, 1996). Maternal cognition was assessed using two tests previously adapted by Prado and colleagues (Prado, Alcock, Muadz, Ullman, & Shankar, 2012) in Indonesia in a maternal supplementation trial, namely, digit span forward and backwards test and mental rotation test. Preschool attendance was assessed by caregiver interview asking whether the child had ever attended preschool.

We adapted all developmental tests and tools to the local setting in Ghana, and the context‐specific criteria were evaluated through two rounds of pilot studies conducted in the study area. Details of the reliability testing and adaptation process have been described elsewhere (Ocansey et al., 2019).

### 2.3 Statistical analysis

Before conducting data analysis, we prespecified decisions regarding the steps for data analysis, calculation of developmental scores, and the list of potential covariates. SAS version 9.4 (SAS Institute, Cary, NC) was used to perform statistical analyses. The SAS GLM procedure was used for multiple linear regression analysis. The level of significance for statistical tests was set at *p* < .05.

For three variables (home inventory, family care indicator, and maternal cognition) collected at follow‐up, 3.0–4.5% of values were missing. We imputed these variables for the children who were in the subsamples but had missing data based on baseline characteristics that were associated with them. We imputed five sets of values for each variable using SAS PROC MI and examined associations between the predictors and outcomes using the imputed covariate values.

For objective 1, to assess the association of linear growth with developmental outcomes, we adapted a method previously described (Li et al., 2004; Pongcharoen et al., 2012) to reduce statistical problems when modelling repeated growth measures such as length or weight across different ages. We conducted four‐stage least squares analyses to estimate the associations of LAZ at birth, and the independent associations of each residual term adjusting for all other growth intervals, with each of the three primary outcomes. In the first three stages, we created three independent variables to represent changes in LAZ from birth to 6 months, 6 to 18 months, and 18 months to 4–6 years by calculating the residuals of LAZ at each time point predicted by linear growth during the previous periods. The fourth stage of the regression approach was a model of the outcome on all three residuals and birth size. For further details, see the supporting information.

Each outcome model was first adjusted for child age at follow‐up and additionally adjusted for baseline covariates (maternal age, education, height, pre‐pregnancy body mass index, marital status, household asset score, household food insecurity score, and nulliparity) and covariates measured after baseline (child sex, intervention group, family care indicators score, data collector, HOME score, maternal depressive symptoms, maternal cognition, and preschool attendance). LAZ at birth and the residuals representing linear growth during the subsequent periods were not collinear by design.

We performed ordinary least squares regression analyses to assess associations between predictors (LAZ at birth and linear growth during each period) and each of the outcomes. Although a unit of a residual is not transparently interpretable, a unit of a delta is easily interpretable as the change in LAZ from one time point to the next. Thus, for each significant residual coefficient in the models defined above, we estimated an additional model using the delta values to interpret the coefficient size. The delta values for each linear growth phase were calculated by subtracting the LAZ of the previous time point from the latter time point. We estimated the change in each outcome for each unit change in LAZ during each linear growth phase, adjusting for LAZ at the beginning of that phase, the age difference between the two LAZ measurements, and covariates measured at baseline and at follow‐up.

For objective 2, to assess the association of Hb concentration with developmental outcomes, we first tested for multicollinearity of the Hb variables across the different ages. The Spearman correlation coefficients for the Hb measurements at 6 months, 18 months, and 4–6 years were in the range of 0.19–0.38, which were below the 0.65 minimum coefficient size we prespecified as likely to cause a problem of multicollinearity in the model. We first performed univariate analysis between each predictor (Hb at 6 months, 18 months, or 4–6 years) and each of the three primary outcomes, first adjusting only for child age in minimally adjusted models and then including other covariates in fully adjusted models. Finally, we ran a full model with all Hb time points in minimally adjusted and fully adjusted analyses for each of the three outcomes as previously described for the linear growth models.

## 3 RESULTS

Table 2 displays characteristics of women enrolled in the original trial and in the two subsamples at baseline and follow‐up. Children in the LAZ and Hb subsamples differed significantly in three background characteristics from those excluded from the analyses. Mothers of children included in the LAZ analyses were less likely to be nulliparous (30.1 vs 38.0%) and more likely to be older (27.1 vs 26.2 years) at enrolment. Children in the Hb subsample were less likely to be nulliparous (31.0 vs 36.3%; Table S1).

**Table 2:** Selected characteristics of women and children in the full iLiNS Dyad Ghana sample and the two subsamples analysed in this study

Variable	Full sample enrolled into original trial	LAZ subsample mean ± *SD* [*n*] or % [*n*/total]	Hb subsample mean ± *SD* [*n*] or % [*n*/total]
Baseline maternal characteristics			
Age (year)	26.7 ± 5.5 [1,320]	27.1 ± 5.5 [710]	27.0 ± 5.5 [617]
Estimated pre‐pregnancy BMI^a^ (kg/m^2^)	24.5 ± 4.4 [1,291]	24.6 ± 4.5 [710]	24.5 ± 4.4 [617]
Gestational age at enrolment (week)	16.1 ± 3.3 [1,311]	16.1 ± 3.3 [710]	16.0 ± 3.3 [617]
Education (year)	7.6 ± 3.6 [1,320]	7.6 ± 3.5 [710]	7.7 ± 3.4 [617]
Haemoglobin concentration (g/dL)	11.1 ± 1.2 [1,319]	11.2 ± 1.2 [710]	11.2 ± 1.2 [617]
Household asset index^b^	0.00 ± 1.0 [1,282]	0.02 ± 0.96 [708]	0.00 ± 0.94 [616]
Household food insecurity index^c^	2.6 ± 4.3 [1,283]	2.5 ± 4.1 [710]	2.6 ± 4.1 [617]
Nulliparous women (%)	33.8 [446/1,320]	30.1 [214/710]	30.9 [191/617]
Gestational age at delivery (week)	39.2 ± 2.0 [1,243]	39.4 ± 1.7 [710]	39.3 ± 1.8 [617]
Characteristics collected after baseline			
Male child (%)	49.0 [611/1,248]	48.0 [341/710]	46.8 [289/617]
Birth weight (g)	2,981 ± 432 [1,158]	2,997 ± 425[710]	2,976 ± 424 [602]
Weight‐for‐age *z*‐score at birth	−0.7 ± 1.0 [1,153]	−0.6 ± 1.0 [710]	−0.7 ± 1.0 [602]
Birth length (cm)	48.4 ± 2.0 [1,153]	48.4 ± 1.9 [710]	48.4 ± 1.9 [599]
Length‐for‐age *z*‐score at birth	−0.7 ± 1.0 [1,153]	−0.6 ± 1.0 [710]	−0.7 ± 1.0 [599]
Length‐for‐age *z*‐score at 6 months	−0.7 ± 1.0 [1,053]	−0.7 ± 1.0 [710]	−0.7 ± 1.0 [609]
Length‐for‐age *z*‐score at 18 months	−0.8 ± 1.0 [1,039]	−0.9 ± 1.0 [710]	−0.9 ± 1.0 [615]
Height‐for‐age *z*‐score at 4–6 years	‐	−0.6 ± 0.9 [710]	−0.6 ± 1.0 [568]
Haemoglobin concentration (g/dL) at 6 months	11.3 ± 1.0 [836]	11.3 ± 1.0 [642]	11.3 ± 1.0 [617]
Haemoglobin concentration (g/dL) at 18 months	11.2 ± 1.1 [794]	11.2 ± 1.1 [669]	11.2 ± 1.1 [617]
Haemoglobin concentration (g/dL) at 4–6 years	‐	11.1 ± 1.1 [630]	11.1 ± 1.0 [617]
Child age (years)	‐	4.9 ± 0.6 [710]	4.9 ± 0.6 [617]
Weight (kg) at 4–6 years	‐	16.5 ± 2.2 [710]	16.4 ± 2.1 [616]
Weight‐for‐age *z*‐score at 4–6 years	‐	−0.7 ± 0.8 [710]	−0.7 ± 0.8 [615]
Height (cm) at 4–6 years	‐	106.2 ± 5.5 [710]	106.2 ± 5.4 [616]
Preschool attendance at 4–6 years	‐	96.3 [684/710]	96.0 [592/617]
Home inventory score at 4–6 years	‐	28.1 ± 4.5 [710]	28.1 ± 4.5 [617]
Maternal depressive symptoms score at 4–6 years	‐	5.7 ± 5.7 [710]	5.9 ± 5.8 [617]
Maternal cognitive *z*‐score at 4–6 years	‐	0.02 ± 0.7 [710]	0.03 ± 0.7 [617]

Abbreviations: BMI, body mass index; iLiNS, International Lipid‐based Nutrient Supplement.

a Estimated pre‐pregnancy BMI was calculated from estimated pre‐pregnancy weight (based on polynomial regression with gestational age, gestational age squared, and gestational age cubed as predictors) and height at enrolment.

b Proxy indicator for household socio‐economic status constructed for each household based on ownership of a set of assets (radio, television, etc.), lighting source, drinking water supply, sanitation facilities, and flooring materials. Household ownership of this set of assets is combined into an index (with a mean of zero and standard deviation of one) using principal components analysis (Vyas & Kumaranayake, 2006). Higher value represents higher socio‐economic status.

c Proxy indicator for household socio‐economic status constructed for each household based on a set of nine questions indicating whether household members experienced food insecurity in the previous 4 weeks (Coates, Swindale, & Bilinsky, 2007). The higher the score, the higher the degree of household food insecurity.

Table 3 shows the estimates of the association of LAZ at birth, and changes in LAZ from birth to 6 months, 6 to18 months, and 18 months to 4–6 years, with each developmental score at age 4–6 years. In a four‐stage least squares analysis, a 1 *SD* difference in LAZ at birth was significantly positively associated with a 0.12 *SD* difference in cognitive scores when controlling for child age at follow‐up (95% CI = 0.05, 0.19, 0.001). In the fully adjusted model, the coefficient was similar (β = 0.10, 95% CI = 0.03, 0.17, 0.005). Change in LAZ from 6 to 18 months was significantly positively associated with cognitive scores at 4–6 years when controlling for child age (β = 0.16, 95% CI = 0.04, 0.28, 0.011) and for covariates measured at baseline and at follow‐up (β = 0.12, 95% CI = 0.00, 0.24, 0.047). Changes in LAZ from birth to 6 months and from 18 months to 4–6 years were not significantly associated with any outcome measured at 4–6 years. In the additional model to interpret the coefficient size, a 1 *SD* difference in ΔLAZ from 6 to 18 months was associated with a difference of 0.15 *SD* in cognitive scores at 4–6 years (95% CI = 0.03, 0.27, 0.017) after adjustment for 6‐month LAZ, the difference in the child's age between the 6 and 18 months linear growth measurements, and child age at follow‐up. In the fully adjusted model, the coefficient size was similar (β = 0.11, 95% CI = 0.00, 0.21, 0.042). The independent variables for the LAZ time points in the model accounted for 2.6% of the variation in cognitive *z*‐scores at age 4–6 years in this cohort. We found no evidence of associations of LAZ at birth and linear growth during any of the three periods (from birth to 6 months, 6 to 18 months, or 18 months to 4–6 years) with motor or social–emotional difficulties scores at 4–6 years (*p* > .05).

**Table 3:** Associations between length‐for‐age *z*‐scores and development at 4–6 years

Variable	Adjusted for child age at follow‐up β (95% CI)	Adjusted for baseline and other covariates^a^ β (95% CI)	*R* ^2^ for the model adjusted for baseline and other covariates
Cognitive factor z‐score (*N* = 701)			
LAZ at birth	0.12 (0.05, 0.19)^c^	0.10 (0.03, 0.17)^c^	0.17
Linear growth birth to 6 months	0.00 (−0.09, 0.09)	0.05 (−0.04, 0.14)	
Linear growth 6 to 18 months	0.16 (0.04, 0.28)^b^	0.12 (0.00, 0.24)^b^	
Linear growth 18 months to 4–6 years	0.08 (−0.05, 0.21)	−0.02 (−0.14, 0.11)	
Motor z‐score (*N* = 710)			
LAZ at birth	−0.02 (−0.09, 0.05)	−0.03(−0.11, 0.04)	0.08
Linear growth birth to 6 months	−0.02 (−0.11, 0.07)	−0.02 (−0.11, 0.08)	
Linear growth, 6 to 18 months	−0.08 (−0.21, 0.05)	−0.06 (−0.19, 0.07)	
Linear growth 18 months to 4–6 years	0.03 (−0.10, 0.16)	0.04 (−0.10, 0.18)	
Social‐emotional difficulties *z*‐score (*N* = 710)			
LAZ at birth	0.02 (−0.06, 0.09)	0.03 (−0.04, 0.10)	0.24
Linear growth birth to 6 months	0.01 (−0.09, 0.11)	−0.05 (−0.14, 0.04)	
Linear growth 6 to 18 months	−0.03(−0.17, 0.10)	−0.02 (−0.15, 0.10)	
Linear growth 18 months to 4–6 years	−0.07(−0.22, 0.07)	0.04 (−0.09, 0.18)	

Abbreviations: BMI, body mass index; HOME, Home Observation for the Measurement of the Environment; LAZ, length‐for‐age *z*‐score.

a Adjusted for child age, sex, nulliparity, data collector, intervention group, baseline maternal age, education, height, pre‐pregnancy BMI, marital status, household asset score, household food insecurity index, family care indicator score at 18 months, HOME score, preschool attendance, maternal depressive symptoms and maternal cognition at follow‐up.

b *p* < .05.

c *p* < .01.

Table 4 presents the estimates of the association of Hb concentration during early life (6 and 18 months), as well as concurrent Hb concentration, with each developmental score at 4–6 years. Adjusting for child age at developmental assessment, Hb concentration at 18 months, but not Hb concentration at 6 months or 4–6 years, was associated with cognitive scores. A 1 g/dL increase in Hb concentration at 18 months was associated with a 0.13 *SD* increase in cognitive scores at 4–6 years when controlling for child age at follow‐up (95% CI = 0.06, 0.20, 0.0003) and for other covariates collected at baseline, and follow‐up (β = 0.11, 95% CI = 0.04, 0.17, 0.001) in the single‐predictor model. When adjusting for Hb at other time points, the association of 18‐month Hb with cognitive scores remained similar in magnitude across age‐adjusted (β = 0.15, 95% CI = 0.08, 0.23, *p* < .0001) and fully adjusted (β = 0.14, 95% CI = 0.07, 0.21, 0.0002) models and remained significant. When including all Hb time points in the model, 6‐month Hb was significantly negatively associated with cognitive scores. The independent variables for the Hb time points in the model accounted for 2.1% of the variation in cognitive *z*‐scores at age 4–6 years in this cohort. We found no evidence of associations of Hb concentration across the different ages with motor or social–emotional difficulties scores at age 4–6 years.

**Table 4:** Associations between haemoglobin concentration and development at 4–6 years

Variable	Each Hb time point entered in separate models		All Hb time points entered in one model		
	Adjusted for child age at follow‐up β (95% CI)	Adjusted for baseline and other covariates^a^ β (95% CI)	Adjusted for child age at follow‐up β (95% CI)	Adjusted for baseline and other covariates^a^ β (95% CI)	*R* ^2^ for the model adjusted for baseline and other covariates
Cognitive factor *z*‐score (*N* = 611)					
Haemoglobin at 6 months	−0.00 (−0.07, 0.07)	−0.02 (−0.09, 0.05)	−0.07 (−0.14,0.01)	−0.07 (−0.15, −0.00)^b^	0.21
Haemoglobin at 18 months	0.13 ± (0.06, 0.20)^c^	0.11 ± (0.04, 0.17)^c^	0.15 ± (0.08,0.23)^d^	0.14 (0.07, 0.21)^d^	
Haemoglobin at 4–6 years	0.02 (−0.05, 0.08)	0.00 (−0.07, 0.06)	0.00(−0.07, 0.07)	0.00 (−0.08, 0.05)	
Motor z‐score (*N* = 617)					
Haemoglobin at 6 months	−0.06 (−0.14, 0.01)	−0.03 (−0.11, 0.05)	−0.04 (−0.12, 0.04)	−0.01 (−0.09, 0.07)	0.08
Haemoglobin at 18 months	−0.06 (−0.14, 0.01)	−0.05 (−0.12, 0.02)	−0.04(−0.12, 0.04)	−0.04 (−0.12, 0.04)	
Haemoglobin at 4–6 years	−0.04 (−0.10, 0.03)	−0.03 (−0.10, 0.04)	−0.02 (−0.09, 0.05)	−0.02 (−0.09, 0.05)	
Social‐emotional difficulties z‐score (*N* = 617)					
Haemoglobin at 6 months	−0.02 (−0.10, 0.06)	−0.00 (−0.08, 0.07)	0.00 (−0.09, 0.09)	−0.01 (−0.09, 0.08)	0.23
Haemoglobin at 18 months	−0.05 (−0.13, 0.03)	−0.01 (−0.08, 0.07)	−0.05 (−0.13, 0.04)	−0.01 (−0.09, 0.07)	
Haemoglobin at 4–6 years	−0.01 (−0.09, 0.07)	0.02 (−0.05, 0.09)	−0.00 (−0.08, 0.08)	−0.02 (−0.05, 0.09)	

Abbreviation: HOME, Home Observation for the Measurement of the Environment.

a Adjusted for child age, sex, nulliparity, data collector, intervention group, baseline maternal age, education, height, pre‐pregnancy BMI, marital status, household asset score, household food insecurity index, family care indicator score at 18 months, HOME score, preschool attendance, maternal depressive symptoms, and maternal cognition at follow‐up.

b *p* < .05.

c *p* < .01.

d *p* < .001.

## 4 DISCUSSION

The present study assessed the association of linear growth during four phases of development (prenatal, from birth to 6 months, from 6 to 18 months, and from 18 months to age 4–6 years) as well as Hb concentrations measured during the first 2 years (6 and 18 months) and later childhood (4–6 years) with cognitive, motor, and social–emotional development at age 4–6 years in a longitudinal cohort in Ghana. Cognitive development at age 4–6 years was associated with LAZ at birth and with linear growth during the 6‐ to 18‐month period but not during the periods from birth to 6 months or 18 months to 4–6 years. Hb at 18 months, but not at 6 months or 4–6 years, was positively associated with cognitive scores. Although Hb at 6 months was not associated with cognitive scores in bivariate analysis, this association was negative when adjusting for Hb at other time points. Motor and social–emotional behaviour scores at age 4–6 years were not associated with LAZ at birth, postnatal linear growth, or Hb concentrations at any time point measured.

Our results are consistent with findings from nine previous studies in LMICs that found stronger associations of linear growth during early childhood before age 1–2 years as compared with later childhood with later cognition or school achievement (Adair et al., 2013; Cheung & Ashorn, 2010; Crookston et al., 2013; Glewwe et al., 2001; Hamadani et al., 2014; Kowalski et al., 2018; Kuklina et al., 2006; Li et al., 2004; Pongcharoen et al., 2012). Together with these studies, our results suggest that during the period from gestation up to age 2 years, exposure to biological and environmental risk factors, as reflected by poor linear growth, is likely to constrain cognitive development with long‐term consequences. In contrast, findings from three other studies indicate that linear growth during later childhood as compared with early childhood is more strongly associated with cognitive development (Fink & Rockers, 2014; Gandhi et al., 2011; Teivaanmäki et al., 2017). This suggests that exposure to biological and environmental risks after age 2 years can have long‐term consequences for cognition and school achievement in certain settings. Further follow‐up of longitudinal cohorts across a range of ages and settings is needed to systematically investigate how contextual factors influence the timing of environmental susceptibility.

In our cohort, we did not find evidence of associations of linear growth during any period of development with fine motor dexterity at age 4–6 years; however, positive associations with motor development at 18 months were reported previously in this same cohort by Prado et al. (2016). Evidence from an earlier study in Guatemala also supports a stronger link between linear growth faltering and motor development at age 24 months in the earlier rather than later infant/toddler period (Lasky et al., 1981). In a recent systematic review and meta‐analysis of associations between linear growth and child development, Sudfeld et al. (2015) reported positive associations between early linear growth status and both early and later childhood motor developments. One potential reason why exposure to risk factors such as linear growth faltering during the first 2 years was associated with early rather than later motor development in our cohort is that the rapid acquisition of motor skills occurs early in life. Motor development before age 2 years may be a more sensitive indicator of developmental status as compared with fine motor dexterity at preschool age, which varies between children but is not a rapidly developing skill at this age (Poole, Miller, & Church, 2005). It is also possible that children who were behind their peers in motor development at an early age caught up in motor skills as they matured (Piek, Dawson, Smith, & Gasson, 2008).

In the present study, we did not find evidence of associations between linear growth or Hb and social–emotional development at preschool age. This is consistent with the lack of association between linear growth and social–emotional development at age 18 months as reported by Prado et al. (2016). The risk factors causing growth stunting or anaemia during those periods may be different from those that are likely to have effects on social–emotional development, such as nurturing and stimulation from the home environment. Similar to our findings, a longitudinal study in China showed no significant associations of birth weight or postnatal linear growth with internalizing, externalizing, or other behavioural problems based on the Child Behavior Checklist at age 4–7 years (Huang, MArtorell, Ren, & Li, 2013). In contrast to our findings, in a study in Belarus, prenatal and early infancy growth but not growth from 1 to 5 years among normal healthy children was associated with reduced externalizing behaviours on the strengths and difficulties questionnaire at age 6.5 years (Yang et al., 2011). Aubuchon‐Endsley et al. (2013) also found positive associations between Hb at 6 months and behaviour (alertness and responsiveness) at age 9 months in Ethiopian children.

Neither linear growth from birth to 6 months of age nor Hb at age 6 months positively predicted later cognitive development in this cohort. A potential reason for this lack of association could be that early linear growth and Hb were not accurate indicators of exposure to environmental insults in our cohort during this period. Heterogeneity in the early postnatal growth rate of infants, including the phenomena of “catch‐up” and “catch‐down” growth in the first 4 months (Mei, Grummer‐Strawn, Thompson, & Dietz, 2004), is relevant to this question. On average, LBW infants grow more rapidly than normal weight infants (a type of catch‐up growth; Brandt, Sticker, Gausche, & Lentze, 2005; Dewey, 1998), and large infants exhibit what has been called catch‐down growth, meaning that they grow less rapidly than normal weight infants. This variability may overshadow other nutritional or infectious factors influencing linear growth in the first 6 months of life, making growth from birth to 6 months a less sensitive marker of environmental insults. A second potential reason for the lack of association between linear growth during birth to 6 months and later development could be that growth during this period is less likely to be influenced by micronutrient deficiencies than is the case during other periods. For some growth‐related nutrients, such as zinc, the infant may be relatively protected from deficiency for the first few months because of nutrient stores at birth. Similarly, Hb at 6 months is strongly influenced by iron stores at birth, which can protect the infant from iron deficiency for 4–8 months (Dewey & Chaparro, 2007). At the same time, the rates of breastfeeding in LMICs, including in our sample, are generally high in the first 6 months (Lancet, 2016), and breastfeeding confers some protection against pathogens in the infant's environment that may compromise growth, Hb, and development.

After 6 months, the protective effects of breastfeeding begin to decline as infants become more exposed to environmental contamination including unhygienic or unsafe and nutritionally inadequate complementary foods, especially in at‐risk populations (Shrimpton et al., 2001). In our sample, anaemia prevalence increased from 35.3% at age 6 months to 42.9% at age 18 months (Adu‐Afarwuah et al., 2019). Additionally, the achievement of certain motor milestones such as sitting and crawling increases the risk of exposure to environmental pathogens. In our sample, the prevalence of inflammation (elevated AGP) increased from 18.8% at age 6 months to 26.8% at age 18 months (Adu‐Afarwuah et al., 2019). Children's exploration of their environment plays an important role in cognitive development and may be a mediating factor in effects of undernutrition and illness. Similarly, iron‐deficiency anaemia is generally more common at age 12 to 18 months than in the first six postnatal months, which could explain the observed positive association between later cognitive scores and Hb at 18 months but not 6 months. After 6 months of age, the infant is more susceptible to iron deficiency because iron stores may be depleted and intake and/or absorption of iron from dietary sources are often insufficient to provide an adequate supply of iron to host tissues including the central nervous system (Krebs & Hambidge, 2007). Together, these changes that occur after age 6 months may make development during the period from age 6 to 18 months more sensitive to long‐term environmental insults.

The observed results for Hb and development in our study are similar to findings from a study in Kosovo conducted by Wasserman and colleagues (Wasserman et al., 1992). In that study, Hb concentrations at 12 and 18 months were more strongly associated with mental development at age 2 years than was concurrent Hb. At follow‐up at age 4 years, 6‐month Hb was negatively associated with IQ, whereas 36‐month Hb was positively associated with IQ (Wasserman et al., 1994). This pattern is similar to our finding of a negative association between 6‐month Hb and cognitive scores when adjusting for Hb at other time points.

Neither change in linear growth from 18 months to 4–6 years nor Hb at age 4–6 years were associated with cognition at 4–6 years, which suggests that the brain may not be as vulnerable during this period to environmental exposures that cause both linear growth faltering or low Hb and long term cognitive deficits as compared with the first 1,000‐day window. The relatively slower rate of some neurodevelopmental processes and reduced demand for certain nutrients after the critical first 1,000‐day window (Cusick & Georgieff, 2016) may explain why exposure to risk factors likely to constrain linear growth may not be as influential during this period.

One strength of this study was our ability to estimate differences in cognitive, motor, and social–emotional functions associated with longitudinal measures of Hb concentration as well as growth trajectories involving estimates of the changes between multiple linear growth time points during childhood. Additionally, we controlled for selected sociodemographic and environmental factors that could potentially confound results including baseline maternal education level, household socio‐economic status, food insecurity, and nulliparity as well as gender, caregiver nurturing and stimulation at home, maternal cognition, and maternal depressive symptoms scores at follow‐up. Finally, we addressed methodological issues caused by the correlation between birth length and postnatal linear growth measures. One limitation of this study is that data collection on linear growth during the main trial was completed when children exited the study at 18 months; thus, we could not examine growth faltering during subintervals between 18 months and 4–6 years in this cohort. Additionally, we recognize the potential for selection bias due to substantial loss to follow‐up caused by missing data. Nonetheless, most maternal baseline characteristics were similar between the LAZ and Hb subsamples and those excluded from the analyses, suggesting a low chance of bias. We also could not adjust for genetic potential and other factors such as children's health condition at follow‐up.

In conclusion, findings of the present study add to the evidence base from LMICs showing positive associations between linear growth and Hb concentration before age 2 years and cognitive but not motor or social‐emotional development in later childhood. Growth and Hb after age 18 months in this cohort were not associated with later cognition, motor, or social‐emotional functions. Although these results support the importance of the first 1,000 days, only a small proportion of the variance in cognitive scores was attributable to linear growth or Hb concentrations in this cohort, suggesting that other genetic and environmental factors unmeasured in this study play a large role. Further research on identifying and addressing those factors is needed.

## CONFLICTS OF INTEREST

The authors declare that they have no conflicts of interest.

## CONTRIBUTIONS

MEO, SA‐A, KGD, and EP designed the research; MEO, SA‐A, MA, SMK, HO, SMT, BMO, and ELP conducted the research; RYY, ELP, and MEO performed the statistical analysis; KGD advised on the analysis; MEO, ELP, and KGD wrote the manuscript; and SA‐A, MA, SMK, HO, BMO, RYY, and SMT reviewed the draft manuscript. All authors critically commented on drafts and approved the final manuscript.

## Supporting information

Table S1:
Background characteristics of children included vs excluded from LAZ and Hb analysis at follow‐up
