# Supplementary material for: The association of early linear growth and haemoglobin concentration with later cognitive, motor, and social–emotional development at preschool age in Ghana
Source: Matern Child Nutr. 2019 Jun 13;15(4):e12834. doi: 10.1111/mcn.12834 (PMC6852555; doi:10.1111/mcn.12834)
Supplement: Supplementary file 1 — Table S1: Background characteristics of children included vs excluded from LAZ and Hb analysis at follow‐up [file MCN-15-e12834-s001.docx]

Online Supporting Material: The Association of Early Linear Growth and Hemoglobin Concentration with Later Cognitive, Motor and Social-emotional Development at Preschool Age in Ghana

**Supplemental Methods**

*Developmental assessments*

*Selection and adaptation of developmental tests and tools*

We selected a set of motor, cognitive and social-emotional development tests according to the following criteria:

1. High priority criteria: a) ability to distinguish between different children’s abilities, resulting in a good distribution of scores in a sample of children at the target age; b) appropriate for the local setting based on children’s familiarity with test materials and procedures; c) high reliability and validity in the local setting; d) does not require extensive training or subjective judgments by the data collectors; e) relatively brief to administer.

2. Medium priority criteria: a) shown to be sensitive to maternal and child nutritional deficiency in previous studies; b) measuring a wide range of abilities that develop during early childhood, if possible tied to brain systems and mechanisms; c) predicts future outcomes, such as later learning disabilities, cognitive scores, school performance, grade level attained in school or skilled employment, etc.

3. Low priority criteria: a) have established norm values for the target age.

We first developed forms and manuals for each test. With five local field workers, we reviewed each test in the original English form, translated items and instructions into three common local languages (Krobo, Ewe, Twi), and trained on instructions and procedures for assessments. We also discussed appropriate materials and pictures to be used for assessments that are common or normal to the local area.

The pilot study team consisted of an international developmental psychologist, a doctoral student researcher trained in child neurobehavioral assessments (field supervisor), and five trained field workers recruited from the study area who were proficient in the local languages spoken in the area. The process was iterative, involving review and modifications of test materials and pictures to identify cultural appropriateness and acceptable level of difficulty, and personalized interviews with mothers of preschool children in the local area to modify forms to be relevant to children in the local context. For the initial round of pilot testing, test-retest reliability ranged from r = 0.64 - 0.87, with the exception of fine motor and executive function tests and parental reports on social-emotional function (r = 0.34 - 0.58). Internal reliability from Cronbach’s alpha ranged from 0.61 - 0.90 and most tests correlated with age, except for the social-emotional scales. After additional modifications, in the final round of pilot testing, reliability of tests of fine motor and executive function improved (r = 0.61- 0.80) and parent reports on children’s social-emotional function showed higher reliability (r = 0.75 - 0.94).

*Description of sub-tests included in cognitive factor analysis*

Language ability was assessed by the Developmental Neuropsychological Assessment (NEPSY II) body part naming and identification and comprehension of instructions subtests (Brooks et al., 2009). Pre-academic skills were assessed using the Parent’s Evaluation of Developmental Status (PEDS) developmental milestones test (Brothers et al., 2008), and executive function was assessed using the head-toe test (Pisani et al., 2015) and a visual search task adapted from the NEPSY (Prado et al., 2012). The block design test, based on the British Ability Scales (BAS II) pattern construction subtest (Elliott et al., 1996) and the Wechsler Primary and Preschool Scale of Intelligence (WPPSI) block design subtest (Wechsler, 2002), was used to assess visuospatial ability. Declarative memory was assessed using a paired associate memory task (Baddeley et al., 1995).

Developmental assessments were conducted by five trained data collectors who were required to pass knowledge and practice-based evaluations before testing or interviewing participants. Details of inter-scorer agreements and test-retest reliability have been reported elsewhere (Ocansey et al., 2019).

*Other covariates collected at follow-up*

*Maternal Cognition*

*Digit span forward and backward tests*

The digit span forward test measures short-term retention and attention and the digit span backward measures the ability to manipulate and retain information in one’s working memory. In both of these tests, increasingly longer sequences of numbers were read out to mothers to either repeat them (digit span forward) or repeat them backwards (digit span backward), until they were incorrectly mentioned on two consecutive trials of the same length (Prado et al., 2012). For each test, we calculated scores as the total number of sequences repeated without errors.

*Mental rotation test*

Mental rotation test measures dynamic mental imagery and visuospatial ability. Mothers were shown five rows of figures and a target figure. They were instructed to mark the figures that were rotations but not mirror images of the target figure (Prado et al., 2012). We calculated scores as the percent of figures correctly marked as rotations.

*Statistical analysis*

*Multi-stage least squares regression analysis*

Prenatal linear growth was represented by LAZ at birth. In the first stage, to calculate the residual R1, we estimated a linear regression model with LAZ at birth predicting LAZ at 6 months. R1 was then outputted and saved from this model as the difference between the predicted LAZ at 6 months and the observed LAZ at 6 months. In the 2^nd^ stage, to calculate the residual R2, we estimated a linear regression model with LAZ at birth and R1 predicting LAZ at 18 months. R2 (difference between predicted LAZ at 18 months and the observed LAZ at 18 months) was outputted and saved from this model. In the 3^rd^ stage, to calculate the residual R3, we estimated a linear regression model with LAZ at birth, R1 and R2 predicting LAZ at 4-6 years. The residual R3 (difference between predicted LAZ at 4-6 years and the observed LAZ at 4-6 years) was outputted and saved from this model. The 4th stage of the regression approach is a model of the outcome on all 3 residuals and birth size. The residuals calculated for each time point can be interpreted as length gain during the specific time periods that is unexplained by previous length measures.

**References for supplemental material**

BADDELEY, A., GARDNER, J. M. & GRANTHAM-MCGREGOR, S. 1995. Cross-cultural cognition: Developing tests for developing countries. *Applied Cognitive Psychology,* 9**,** S173-S195.

BROOKS, B. L., SHERMAN, E. M. & STRAUSS, E. 2009. NEPSY-II: A developmental neuropsychological assessment. *Child Neuropsychology,* 16**,** 80-101.

BROTHERS, K. B., GLASCOE, F. P. & ROBERTSHAW, N. S. 2008. PEDS: developmental milestones—an accurate brief tool for surveillance and screening. *Clinical pediatrics,* 47**,** 271-279.

COATES, J., ANNE SWINDALE AND PAULA BILINSKY 2007. Household Food Insecurity Access Scale (HFIAS) for Measurement of Food Access: Indicator Guide Washington, D.C.

: Academy for Educational Development. Food and Nutrition Technical Assistance Project (FANTA)

ELLIOTT, C. D., SMITH, P. & MCCULLOCH, K. 1996. British Ability Scales second edition (BAS II): administration and scoring manual. *London: NFER-Nelson*.

OCANSEY, M. E., ADU-AFARWUAH, S., KUMORDZIE, S. M., OKRONIPA, H., YOUNG, R. R., TAMAKLOE, S. M., OAKS, B. M., DEWEY, K. G. & PRADO, E. L. 2019. Prenatal and postnatal lipid-based nutrient supplementation and cognitive, social-emotional, and motor function in preschool-aged children in Ghana: a follow-up of a randomized controlled trial. *Am J Clin Nutr,* 109**,** 322-334.

PISANI, L., BORISOVA, I. & DOWD, A. J. 2015. International development and early learning assessment technical working paper. Save the Children.

PRADO, E. L., ALCOCK, K. J., MUADZ, H., ULLMAN, M. T. & SHANKAR, A. H. 2012. Maternal multiple micronutrient supplements and child cognition: a randomized trial in Indonesia. *Pediatrics***,** peds. 2012-0412.

VYAS, S. & KUMARANAYAKE, L. 2006. Constructing socio-economic status indices: how to use principal components analysis. *Health Policy and Planning,* 21**,** 459-468.

WECHSLER, D. 2002. *The Wechsler intelligence scale for children,* San Antonio, TX

The Psychological Corporation

**Supplemental Table**

| **Table S1: Background characteristics of children included vs excluded from LAZ and Hb analysis at follow-up** | | | | | | | |
| --- | --- | --- | --- | --- | --- | --- | --- |
|  | **Children included in LAZ analysis** | **Children excluded from LAZ analysis** |  | **Children included in Hb analysis** | **Children excluded from Hb analysis** | |  |
| Baseline variables | Mean ± SE [n] or % [n/total] | Mean ± SE [n] or % [n/total] | p-value | Mean ± SE [n] or % [n/total] | Mean ± SE [n] or % [n/total] | p-value | |
| Maternal age (yr) | 27.1 ± 0.2[710] | 26.2 ± 0.2[610] | 0.002 | 27.0 ± 0.2[617] | 26.4 ± 0.2[703] | | 0.061 |
| Estimated maternal pre-pregnancy BMI^1^ (Kg/m^2^) | 24.6 ± 0.2[710] | 24.4 ± 0.2[581] | 0.345 | 24.5 ± 0.2[617] | 24.6 ± 0.2[674] | | 0.697 |
| Gestational age at enrolment (wk) | 16.1 ± 0.1[710] | 16.2 ± 0.1[601] | 0.959 | 16.0 ± 0.1[617] | 16.2 ± 0.1[694] | | 0.120 |
| Maternal education (yr) | 7.6 ± 0.1[710] | 7.7 ± 0.1[610] | 0.586 | 7.7 ± 0.1[617] | 7.6 ± 0.1[703] | | 0.520 |
| Maternal hemoglobin concentration (g/dL) | 11.2 ± 0.1[710] | 11.1 ± 0.1[609] | 0.085 | 11.2 ± 0.1[617] | 11.1 ± 0.1[702] | | 0.295 |
| Household asset score^2^ | 0.02 ± 0.04[708] | -0.01 ± 0.04[574] | 0.643 | -0.00 ± 0.04[616] | 0.01 ± 0.04[666] | | 0.747 |
| Household food insecurity index^3^ | 2.5 ± 0.2[710] | 2.7 ± 0.3 [244] | 0.541 | 2.6 ± 0.2 [617] | 2.7 ± 0.2 [666] | | 0.647 |
| Nulliparous women (%) | 30.1[214/710] | 38.0[232/610] | 0.003 | 31.0[191/617] | 36.3[255/703] | | 0.042 |
| Male child (%) | 48.0[341/710] | 43.1[270/538] | 0.450 | 46.8[289/617] | 51.0[322/631] | | 0.139 |
| ^1^Estimated pre-pregnancy BMI was calculated from estimated pre-pregnancy weight (based on polynomial regression with gestational age, gestational age squared, and gestational age cubed as predictors) and height at enrollment. ^2^Proxy indicator for household socioeconomic status constructed for each household based on ownership of a set of assets (radio, television etc.), lighting source, drinking water supply, sanitation facilities, and flooring materials. Household ownership of this set of assets is combined into an index (with a mean of zero and standard deviation of one) using principal components analysis (Vyas and Kumaranayake, 2006). Higher value represents higher socioeconomic status. ^3^Proxy indicator for household socioeconomic status constructed for each household based on a set of nine questions indicating whether household members experienced food insecurity in the previous four weeks (Coates, 2007). The higher the score, the higher the degree of household food insecurity. | | | | | | | |
